# Israel’s First Port-Based Hepatic Artery Infusion Program: Early Outcomes in Colorectal Liver Metastases

**DOI:** 10.1245/s10434-025-17910-9

**Published:** 2025-07-27

**Authors:** Roy Apel, Omri Sulimani, Gali Perl, Chen Abitbol, Yael Berger MD, Shlomit Tamir, Oran Zlotnik, Nasser Abdul Halim, Ana Tovar, Baruch Brenner, Nir Wasserberg, Eran Sadot

**Affiliations:** 1 Department of Surgery, Rabin Medical Center, The Sackler School of Medicine, Tel-Aviv University, Tel Aviv, Israel; 2 Institute of Oncology, Davidoff Center, Rabin Medical Center, The Sackler School of Medicine, Tel-Aviv University, Tel Aviv, Israel; 3 Department of Radiology, Rabin Medical Center, The Sackler School of Medicine, Tel-Aviv University, Tel Aviv, Israel; 4 Department of Pathology, Rabin Medical Center, The Sackler School of Medicine, Tel-Aviv University, Tel Aviv, Israel

**Keywords:** Hepatic artery infusion, Colorectal liver metastases, Reservoir-less port, Feasibility, First Israeli experience

## Abstract

**Background:**

Over the last three decades, hepatic arterial infusion (HAI) chemotherapy combined with systemic therapy has been established as a treatment strategy for patients with unresectable liver metastases. Facilitating a HAI program outside the USA and Europe remains underutilized in regions, including Israel, and requires multidisciplinary collaboration. The purpose of this study is to describe the feasibility and early outcomes of the initiation of the first hepatic artery infusion program in Israel.

**Patients and Methods:**

This retrospective analysis was conducted at a tertiary medical center in Israel. It included 21 patients with unresectable colorectal liver metastases who underwent hepatic artery infusion port placement between February 2020 and March 2022. All participants underwent hepatic artery infusion port placement with most subsequently receiving chemotherapy. The study evaluated perioperative outcomes, complication rates, response rates, and conversion to resectability at 6 months.

**Results:**

Hepatic arterial infusion chemotherapy was successfully administered to 95% of patients. The overall 90-day major complication rate was 24%, with no reported mortality. At 6 months, the partial response rate was 33%, and the disease control rate was 83%. Four out of five patients (80%) with neoadjuvant intent converted from unresectable to resectable during HAI treatment.

**Conclusions:**

This study demonstrates the feasibility and safety of implementing a new HAI program for carefully selected patients with colorectal liver metastases. Despite the cohort being heavily pretreated with systemic chemotherapy, promising hepatic response rates and disease control rates were observed. These findings support the potential for expanding access to HAI therapy in new geographic regions.

Colorectal cancer (CRC) is the third most common diagnosed malignancy in the USA^1^ and it is the fourth leading cause of cancer related deaths in the world.^2^ Approximately 70% of patients with CRC will develop liver metastases, of which 80% are unresectable. Colorectal liver metastases (CRLM) are a unique phenomenon of stage 4 metastatic cancer that can potentially be cured through surgical resection^.^ The 10-year survival rates after CRLM resection are approximately 24%, with 20% rate of cure.^3^

Hepatic artery infusion (HAI)^4^ is a technique of introducing chemotherapy directly into the hepatic arterial circulation used as a treatment strategy for patients with unresectable liver dominant metastasis. Since liver metastases derive most of their blood supply from the hepatic artery, while normal liver tissue is primarily perfused by the portal vein, HAI was developed as a model to ensure greater local concentration of cytotoxic agents.^5,6^

A phase 2 trial of patients with extensive unresectable CRLM showed that 47% were converted from unresectable to complete resection after combining systemic and pump-based HAI FUDR (floxuridine) chemotherapy.^7^ Of note, conversion to complete hepatic resection is one of the most influential prognostic factors for long-term survival.^8,9^ Another study showed that combined systemic and HAI chemotherapy produced a 52% conversion rate, coupled with a 5-year survival rate of 36%, which represents a promising effect compared with modern systemic chemotherapy for unresectable CRLM, with only 12% conversion rate when applied alone.^10^ A recent international multicentric, prospective, phase II trial (OPTILIV)^11^ investigated triplet chemotherapy by port-based HAI (oxaliplatin/5-FU/irinotecan) combined with systemic cetuximab in 64 patients with unresectable RAS-wild-type CRLM after a first-line systemic treatment. Up to three extrahepatic lesions (less than 1 cm) were allowed and occurred in 41% of the patients. Conversion to resection rate was 30% and objective response rate was 41%.^12–15^

Most research regarding HAI therapy has been conducted in North America in the past 40 years, mostly through use of fluorodeoxyuridine (FUDR).^16^ However, its administration is occasionally limited by biliary toxicity.^17,18^ Oxaliplatin-based HAI offers a few advantages. Oxaliplatin is mostly used with reservoir-less ports, which offer a more affordable option, due to both the low price of the medication and the administration device. In comparison, reservoir-less ports cost approximately 150 euros, versus pumps, which cost approximately 10,000 USD.^19^ Furthermore, pumps used to administer FUDR HAI in North America contain Freon gas, which is banned in certain countries for environmental reasons.^20^ Additional advantages of oxaliplatin include preferred accumulation in liver tumors compared with normal hepatocytes (4.3 ratio of tumor/normal liver parenchyma concentration)^21^ with a liver extraction ratio (0.47), which can reduce the systemic side-effects of oxaliplatin (e.g., decreased peripheral neuropathy).^22^ Several studies have previously demonstrated an advantage for reservoir-less ports in terms of tolerability.^20^

This study presents the first Israeli HAI program facilitating reservoir-less port and using oxaliplatin chemotherapy. Considering the limited data regarding HAI treatment programs outside North America and Europe, this study aimed to describe the feasibility and safety of initiating the first HAI program in Israel and to assess surgical outcomes, surgical complications, and early oncological outcomes for colorectal liver metastases.^23,24^

## Patients and Methods

### Patient Selection

The research was conducted in Rabin Medical Center and included all patients who underwent HAI port placement between February 2020 and March 2022. Patient data were retrieved from a prospectively maintained database. Institutional research committee approved the protocol. All were patients with colorectal cancer with preoperative imaging showing liver dominant metastases. Unresectable disease was defined as resection of all three hepatic veins, both portal veins, or the retrohepatic vena cava, or a resection that leaves fewer than two adequately perfused and drained liver segments.^7^ Preoperative imaging included chest abdomen and pelvic computed tomography (CT) or positron emission tomography (PET) CT to evaluate disease burden. Included patients had no signs of cirrhosis or portal hypertension. Patient demographics, clinical history, prior chemotherapy treatment regimens and perioperative outcomes were assessed. A preoperative CT angiogram was performed to evaluate anatomical arterial variations and assure anatomy fit with HAI catheter placement. Patients were selected on the basis of limited extrahepatic disease that was planned for future resection, ablation, or radiation. Postoperative computed tomography angiography to evaluate port location was conducted in all patients during admission.

### Multidisciplinary Team

An institutional team including hepatobiliary surgeons, medical oncologists, interventional radiologists, and nuclear medicine physicians was assembled. HAI chemotherapy is currently administered by two oncologists (B.B., G.P.). This was purposefully limited to maximize comfort with HAI therapy, facilitate proficiency, and minimize treatment variation. All team members received formal training on port placement, treatment protocol, and complication management.

### Surgical Approach

All surgical procedures were performed in Rabin Medical Center by the same leading surgeons (E.S., O.S., O.Z.) with alternating assistants. Procedures either entailed port placement alone or included primary colorectal tumor resection, hepatectomy, and/or liver ablation. The previously described surgical technique^25^ includes inspection of the peritoneal cavity for metastatic disease, isolation and cannulation of the gastroduodenal artery, and ligation of all branches of the gastroduodenal artery. The port is implanted in the subcutaneous tissue in the left lower chest and connected to a catheter. Perfusion test was performed by injecting methylene blue into the port and demonstrating adequate liver only distribution. Cholecystectomy was completed if not previously performed to avoid future complications during HAI delivery.^26^ For this study the Celsite® arterial access port was used in all patients (Celsite T302 catheter, BBraun, Velizy, France).^11^

### Postoperative Procedures and Initiation of HAI Chemotherapy

Postoperative technetium-99-labeled macroaggregated albumin (Tc-99m MAA) scintigraphy was performed to confirm the absence of extrahepatic perfusion. After satisfactory scintigraphy study, chemotherapy given via HAI port was typically administered 1 month post-surgery. Chemotherapy nurses administered the chemotherapy regimens via the port. Planned regimen consisted of oxaliplatin through the port in body surface area (BSA) adjusted administration. Doses of oxaliplatin were 85 mg/m^2^ administered over 3 hours. Simultaneously 5FU was administered for 2 days. One HAI cycle was defined as 15 days followed by a second cycle. Additional systemic chemotherapy regimen was administered per the discretion of medical oncologists.

Postoperative follow-up included periodic clinic visits, record of chemotherapy complications, and laboratory analysis. Laboratory analysis performed every 2 weeks included complete blood count (CBC), liver function tests including enzymes and bilirubin, albumin levels, and lactate dehydrogenase (LDH) levels.

### Feasibility, Safety, and Response Rates

Feasibility was defined as receiving at least one cycle of HAI chemotherapy. Major postoperative complications (Clavien–Dindo classification grade 3 or higher) and specific port-related complications were recorded within 90 days post-surgery. Objective response rates were assessed 3 and 6 months postoperatively using contrast-enhanced CT reviewed by staff radiologists. Objective response rates were evaluated and reported on the basis of Response Evaluation Criteria in Solid Tumors (RECIST) guidelines (version 1.1).^27^ Response rates were calculated by analyzing data from abdominal CT scans conducted prior to the HAI surgery.

### Methods of Measurement

Clinical risk score for colorectal cancer recurrence after resection of hepatic metastases was measured as previously reported, and the high-risk group was defined as clinical risk score ≥ 3.^28^ Extra hepatic disease (EHD) was defined as a disease known at the time of primary surgery.

### Statistical Analysis

Patient demographics and tumor characteristics were reported as counts and percentages for categorical variables and medians with interquartile ranges (IQRs) for continuous variables. Response rates and feasibility outcomes were reported descriptively. All analyses were performed using SPSS software version 26 (IBM Corp., Armonk, NY).

## Results

### Patient Characteristics

During the study period, 21 patients with CRLM underwent HAI port placement. Preoperative evaluation defined patients as resectable or unresectable. Treatment intent was neoadjuvant (i.e., conversion from unresectable to resectable) in five (23.8%) patients and adjuvant in four (19%) patients. Intent was palliative in 12 patients (57.1%). All treatments were given at our institution. The median length of follow-up from HAI port placement was 14 months (IQR 9–16 months).

HAI-port-eligible patients were treated before surgery with at least a first line of systemic chemotherapy with one exception, a patient who presented with unresectable CRLM and was treated with HAI chemo at first line. The first HAI cycle chemotherapy was initiated at a median of 46 days (IQR 35–54 days) after port placement. The median age was 56 years (IQR 39–67 years). Most patients, 19 (90.4%), presented with synchronous disease. In addition, most patients (20 patients, 95.2%) were heavily pretreated with chemotherapy before HAI placement with a median number of chemotherapy cycles of 17.5 (IQR 12–35.2), of whom 7 (33.3%) had already been treated with second-line chemotherapy. The median number of CRLM before HAI placement was 9 (IQR 7–23) with 12 (57.1%) patients having liver metastases bigger than 5 cm. All patients had bilobar liver metastatic disease and six patients (28.5%) had more than ten CRLM at operation time. Primary tumor location was rectal in 11 (52.3%), sigmoid colon in 8 (38%), and right colon in 2 (9.5%) patients. Extrahepatic metastatic disease was present in 7 (33.3%) patients. Ten (47.6%) patients had high clinical risk score (CRS ≥ 3), and mutational analysis results of 20 patients showed 10 (50%) with Ras mutation. Patient characteristics are presented in Table 1.

**Table 1:** Patient and tumor characteristics

Patient characteristic	*N* (%)	Median (IQR)
Total patients	21 (100)	
Sex		
Male	12 (57.1)	
Female	9 (42.9)	
Race, white	21 (100)	
Age (years)		56 (39.5–67.5)
BMI		25.8 (20.8–28.2)
BMI ≤ 30	19 (90.4)	
Synchronous disease	19 (90.4)	
Pre-HAI placement chemotherapy	20 (95.2)	
Number of pre-HAI placement chemotherapy cycles		17.5 (12–35.2)
Pre-HAI placement first line chemotherapy		
FOLFOX + bevacizumab	8 (38)	
FOLFOX + cetuximab	2 (9.5)	
FOLFIRI + panitumumab	3 (14.2)	
FOLFIRI+ bevacizumab	5 (23.8)	
CAPOX+ panitumumab	1 (4.7)	
FOLFIRI	1 (4.7)	
Pre-HAI chemotherapy first line	20 (95.2)	
Pre-HAI chemotherapy second line	7 (33.3)	
No. of CRLM		9 (7–23)
No. of CRLM > 10	6 (28.5)	
Bilobar disease	21 (100)	
Pre HAI placement CEA levels (ng/mL)		36 (4.8–70.5)
Pre-HAI CEA > 50 (ng/mL)	6 (28.5)	
Size of largest metastasis (cm)		5.5 (3.3–8.2)
CRLM size > 5 cm	12 (57.1)	
High clinical risk score*	10 (47.6)	
HAI intent		
Neoadjuvant	5 (23.8)	
Adjuvant	4 (19)	
Palliative	12 (57.1)	
Presence of extrahepatic disease**	7 (33.3)	
Lung	2 (9.5)	
Retroperitoneal lymph nodes	6 (28.5)	
Bone	1 (4.7)	
Adrenal	1 (4.7)	
Primary tumor location	21 (100)	
Rectum	11 (52.3)	
Sigmoid colon	8 (38)	
Right colon	2 (9.5)	
Mutational status		
Kras wild type (10/20 patients***)	10 (50)	
Braf wild type (17/17 patients***)	17 (100)	
EGFR wild type (10/10 patients***)	10 (100)	
MSS Microsatellite instability status (16 out of 16 patients***)	16 (100)	
Preoperative albumin < 3.5	2 (9.5)	

^*^Fong clinical risk score ≥ 3

^**^One patient had two extrahepatic sites, and one patient had three extrahepatic sites.

^***^Total number of patients where result was available.

*HAI* hepatic artery infusion, *BMI* body mass index, *CRLM* colorectal liver metastasis, *FOLFOX* leucovorin, 5FU, oxaliplatin, *FOLFIRI* leucovorin, 5FU, irinotecan, *EGFR* epidermal growth factor receptor, *MSI* microsatellite instability, *MSS* microsatellite stable

### Operative Details and Complications

Table 2 presents the HAI placement perioperative details. HAI operations were combined with hepatectomy in 18 (85.7%) patients (some of these were biopsies of representative lesions) and combined with colectomy or proctectomy in 11 (52.3%) patients. The surgical approach was laparotomy (17 patients, 80.9%), laparoscopic (3 patients, 14.2%), or robotic (1 patient, 4.7%) technique. Arterial anatomy was observed aberrant in 8 patients (38%). Port catheter was inserted through the gastroduodenal artery in all patients.

**Table 2:** Perioperative details of patients undergoing hepatic artery infusion port placement

Perioperative details	*N* (%)	Median (IQR)
Surgical approach	21 (100)	
Open	17 (80.9)	
Laparoscopic	3 (14.2)	
Robotic	1 (4.7)	
Concomitant hepatectomy	18 (85.7)	
Concomitant colectomy/proctectomy	11 (52.3)	
Liver ablation	7 (33.3)	
Intraoperative	6 (28.5)	
Preoperative	1 (4.7)	
Ligation of aberrant artery	5 (23.8)	
Arterial anatomy		
Conventional	13 (61.9)	
Variant	8 (38)	
Accessory left hepatic artery	5 (23.8)	
Accessory right hepatic artery	1 (4.7)	
Replaced left hepatic artery	1 (4.7)	
Replaced right hepatic artery	1 (4.7)	
Operative time (min)		
All cases		319 (250.7–400)
HAI only operation (*n* = 2)		198 (189–207)
HAI and additional procedure (hepatectomy or colectomy) (*n* = 19)		334 (256.7–401.7)
EBL (mL)		50 (20–200)
Blood transfusion	0	
Postoperative hospital stay (days)		7 (6–9)
90-day mortality	0	
Readmission (90 days)	11 (52.3)	
Reoperation	1 (4.7)	
90-day postoperative complications (Clavien–Dindo grade 3 or higher), overall complications	5 (23.8)	
Non-HAI-related	4 (19)	
HAI-related postoperative embolization of replaced right hepatic artery	1 (4.7)	
HAI-related cutaneous port pocket complications	0	
Initiated HAI therapy	20 (95.2)	

The study had no 90-day mortality. Major postoperative complications were noted in five (23.8%) patients, of which one was HAI-specific complication. Non-HAI-related complications included postoperative pneumothorax that required thorax drain insertion, pleural effusion that required thoracocentesis, colorectal anastomotic leak that required relaparotomy, and postoperative cholangitis treated with percutaneous biliary drainage. HAI-specific complication^29^ was noted in one patient who required postoperative embolization of an accessory replaced right hepatic artery that was missed on initial preoperative evaluation but was later identified on routine postoperative CT angiogram. HAI chemotherapy was administered in 20 (95.2%) patients, as 1 patient from a foreign country returned to her home country after surgery. The initial chemotherapy regimen via HAI port was oxaliplatin in 18 patients (85.7%) and mitomycin C in 2 patients (9.5%). Of patients receiving mitomycin C as first-line therapy, one had history of adverse reaction to oxaliplatin infusion and the second showed progression under previous systemic chemotherapy with oxaliplatin.

### Early Oncological Outcomes

**Fig. 1:**
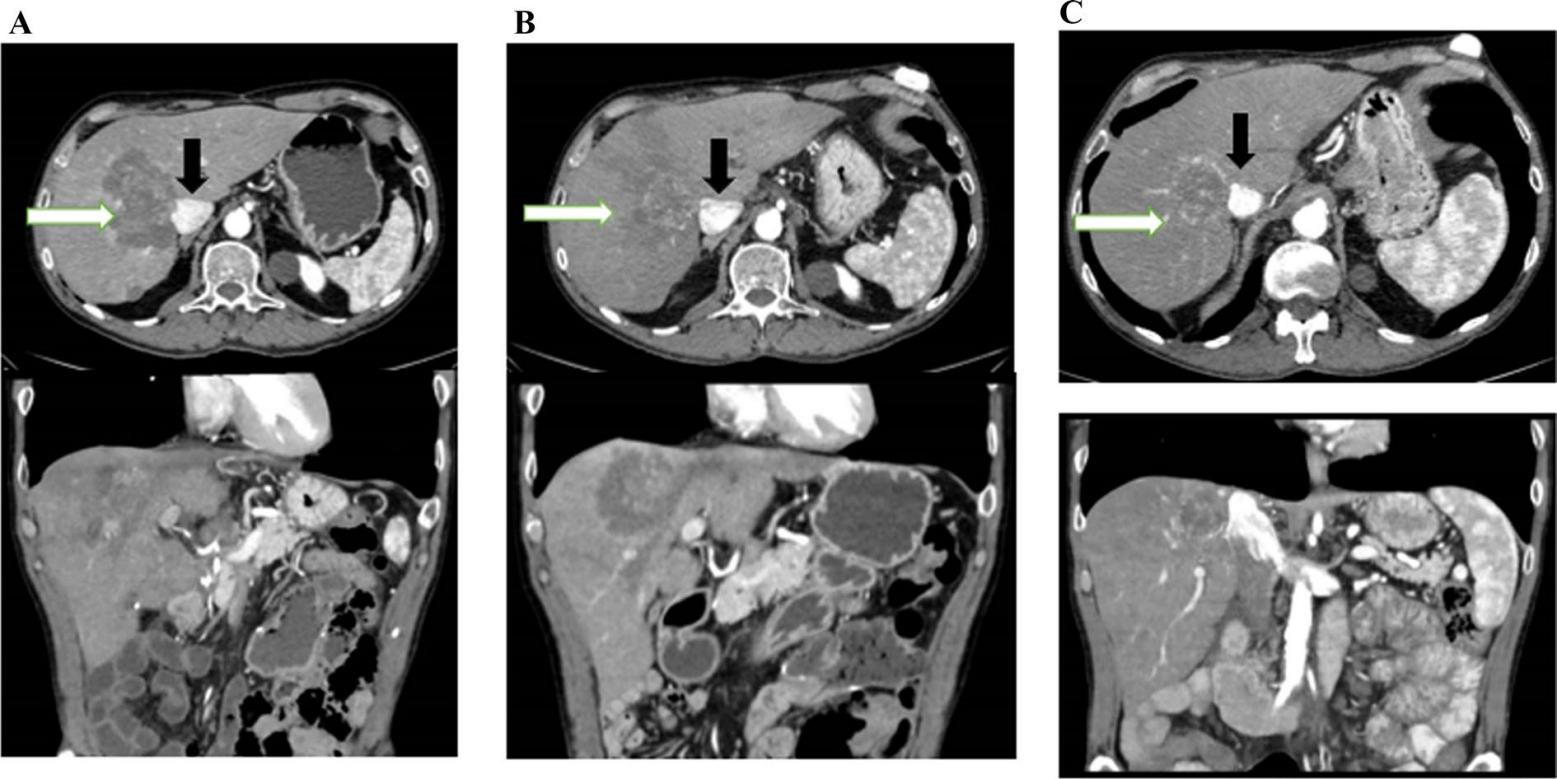
Representative computed tomography (CT) images showing a 60-year-old male patient treated with hepatic artery infusion (HAI) with neoadjuvant intent and after treatment successfully converted to resectable; he was previously treated with 16 cycles of systemic FOLFIRI bevacizumab; white arrow points to lesion, black arrow points to IVC; **A** before HAI, CT shows unresectable disease with tumor involving the IVC; **B** 3 months after HAI port insertion treated with HAI oxaliplatin, corresponding 3-month CT demonstrated decreased IVC involvement; **C** 6 months after HAI port insertion, corresponding 6-month CTs demonstrate continued tumor shrinkage with minimal IVC involvement

A total of 12 patients were eligible for calculation of RECIST criteria at 6-month follow-up. The remaining patients were excluded from objective response rates (ORR) evaluation due to lack of measurable target lesions at baseline, prior complete resection or ablation of liver metastases, or post-embolization changes precluding RECIST-based assessment. The hepatic response rates were reported at 3 months and 6 months after surgery and are presented in Table 3. At 3-month time interval, one (7.6%) patient demonstrated partial response, nine (69.2%) demonstrated stable disease, and three (23%) demonstrated progressive disease. The calculated disease control rate (DCR) at 3 months was 76%. At 6-month time interval, four (33.3%) patients demonstrated partial response, six (50%) showed stable disease, and two (16.6%) demonstrated progressive disease. The calculated 6-month DCR was 83%. Representative CT images from selected patients are shown in Fig. 1.

**Table 3:** Response assessment for eligible patients

RECIST 3-month response rate (*n* = 13)	*N* (%)
Progressive disease	3 (23)
Stable disease	9 (69.2)
Partial response	1 (7.6)
RECIST 6-month response rate (*n* = 12)	
Progressive disease	2 (16.6)
Stable disease	6 (50)
Partial response	4 (33.3)

*RECIST* response evaluation criteria in solid tumors—version 1.1

At the end of follow-up period, 20 patients (95.2%) were alive with disease and 1 patient was with no evidence of disease (NED). Four (80%) of five patients who were treated with neoadjuvant intent converted from unresectable to resectable during the HAI treatment and underwent complete hepatic resection of CRLM. Two of these patients had bilobar CRLM and rectal primaries, and after more than 12 months of combined systemic and HAI chemotherapy, following marked response with only a few residual lesions, underwent parenchyma-sparing liver resection with rectal resection. Another two patients presented with synchronous sigmoid tumors and bilobar CRLM requiring two-stage hepatectomy; one had right lobe lesions involving the IVC. HAI ports were placed during the first-stage surgery and both ultimately completed curative resection. The fifth patient, who had synchronous bilobar CRLM and an obstructing sigmoid tumor, progressed on HAI and systemic treatment and did not undergo resection.

## Discussion

Historically, hepatic arterial infusion chemotherapy treatment has been confined to a limited number of prominent institutions in North America and Europe, thereby restricting access to this effective therapy for the majority of patients.^7,11,30^ There is a paucity of data regarding the global safety implications of expanding this treatment strategy. This study presents the first cohort of patients in Israel to undergo HAI-directed therapy, facilitating an assessment of its safety and feasibility in this population. It outlines the successful implementation of a HAI program and presents outcomes from a series of 21 carefully selected patients with CRLM, 95.2% of whom initiated treatment. The 90-day perioperative major complication rate was 23.8%, with no perioperative mortality observed at 90 days, and a 6-month DCR of 83%.

As mentioned above, our program employs a port-based approach using oxaliplatin or mitomycin C, which differs fundamentally from the pump-based FUDR protocols commonly used in the USA, particularly at MSKCC. This distinction is important, as FUDR—despite its high hepatic arterial exposure—is limited by biliary toxicity and availability issues in many regions. In contrast, oxaliplatin demonstrates favorable tumor uptake, a more favorable toxicity profile, and lower cost, making port-based HAI a practical and scalable alternative.^19^

Our findings are consistent with prior investigations. A study by Creasy from Duke University described their experience initiating a HAI program in a cohort of 21 patients with CRLM.^23^ They reported successful HAI initiation in 20 patients (95%), with no 90-day mortality, an overall surgical morbidity rate of 19%, and a 3-month hepatic DCR of 76%.^23^ Our results compare favorably, demonstrating the safety and feasibility of implementing a new HAI program utilizing a multidisciplinary team approach. In addition, we observed similar short-term outcomes to those reported by the MSKCC group in their investigation of variant arterial anatomy and technical complications associated with HAI placement.^29^

In establishing a new program such as HAI, our initial focus during implementation centered on ensuring safety and demonstrating feasibility. Accordingly, all surgical procedures associated with HAI were conducted by surgeons who possessed substantial experience from previous institutions. We underscore the critical role of a multidisciplinary HAI team in facilitating patient selection, providing perioperative support, and overseeing postoperative management.

Our study population differed from the previously reported cohort from Duke University^23^ in having a higher burden of extrahepatic disease. However, similar to their study, a significant proportion of patients (more than 50%) had experienced treatment failure with first-line therapy, with the remaining having progressed after second-line therapy. These findings suggest that HAI was employed in a population heavily pretreated with systemic chemotherapy. Despite this, a promising partial response rate of 33.3% and a DCR of 83% were observed at 6 months. These outcomes are potentially superior to what could be expected with further lines of systemic therapy alone.

Several limitations were identified in this study. Firstly, it was conducted retrospectively at a large academic medical center, inherently introducing biases that are typical of such study designs. Moreover, the cohort of patients receiving HAI treatment was highly selected, which may not be representative of the broader population of individuals with CRLM. Patients undergoing HAI treatment likely exhibited higher baseline performance status and possessed the social support and ability to travel for frequent medical appointments. Furthermore, the availability of response and outcomes data was limited to a subset of the cohort. Despite these limitations, this study marks a crucial milestone in the safe and successful implementation of the first HAI program in Israel, aimed at broadening access to this treatment modality.

## Conclusions

This study demonstrates the feasibility of implementing a new HAI program and underscores the safe delivery of HAI treatment to carefully selected patients with colorectal liver metastases (CRLM). Establishing such a program at a tertiary referral center necessitates assembling and training a dedicated multidisciplinary team. Despite the early stage of our HAI program, the low and acceptable rates of complications observed in this study, alongside promising hepatic response rates and DCR, are encouraging. These outcomes are noteworthy in a cohort of patients who have undergone extensive prior chemotherapy treatments.
